# Relative bed allocation for COVID-19 patients, EHR investments, and COVID-19 mortality outcomes

**DOI:** 10.1371/journal.pone.0286210

**Published:** 2023-10-26

**Authors:** Pankaj C. Patel, Mike G. Tsionas, Srikant Devaraj

**Affiliations:** 1 Villanova School of Business, Villanova University, Villanova, Pennsylvania, United States of America; 2 Montpellier Business School, France and Lancaster University Management School, Lancaster, United Kingdom; 3 Center for Business and Economic Research, Miller College of Business, Ball State University, Muncie, Indiana, United States of America; University of Georgia, UNITED STATES

## Abstract

Managing flexibility in the relative bed allocation for COVID-19 and non-COVID-19 patients was a key challenge for hospitals during the COVID-19 pandemic. Based on organizational information processing theory (OIPT), we propose that the local electronic health record (EHR) systems could improve patient outcomes through improved bed allocation in the local area. In an empirical analysis of county-level weekly hospital data in the US, relative capacity of beds in hospitals with higher EHR was associated with lower 7-, 14-, and 21-day forward-looking COVID-19 death rate at the county-level. Testing for cross-state variation in non-pharmaceutical interventions along contiguous county border-pair analysis to control for spatial correlation varying between state variations in non-pharmaceutical intervention policies, 2SLS analysis using quality ratings, and using foot-traffic data at the US hospitals our findings are generally supported. The findings have implications for policymakers and stakeholders of the local healthcare supply chains and EHR systems.

## Introduction

During COVID-19, hospitals in the US have faced increased pressure to manage their limited bed capacity [1, 2]. Bed capacity is sticky in the short run, requiring hospitals to engage in operational flexibility by delaying, postponing, or vetting through non-COVID-19 patients with the most urgent needs [3–5]. The operational flexibility is reflected in the relative allocation of beds between COVID-19 and non-COVID-19 patients [referred to as “relative capacity” for this study]. Local coordination among health providers could be a pivotal element to helping manage bed capacity and improving the flow of patients through shared resources, information, and knowledge [6–9]. Electronic health records (EHR) systems may therefore provide the necessary circuitry for coordination [8] as hospitals manage bed capacity to improve COVID-19 mortality.

EHR investments were crucial to coordination. Studies have demonstrated that the presence of “EHR-derived information about patients’ disease condition, treatments, interventions, clinical exams with other data sources is of paramount importance for a deeper comprehension of the COVID-19 disease mechanism and severity manifestation” [10, p. 9]. Facing a variety of challenges during COVID-19, the vertical and lateral fit enhanced by EHR could improve administrative and clinical responses. As the hospital copes with the need to manage COVID-19 and non-COVID-19 health care demands, the degree of EHR adoption may facilitate improved management of uncertainty and complexity to lower mortality, managing length of stay, and readmissions. EHR provides the necessary infrastructure to improve administrative and clinical capabilities in times of COVID-19 [11–14]. Inter- and intra-hospital coordination supported by EHR could lower local death rates of COVID [15–17].

**Fig 1:**
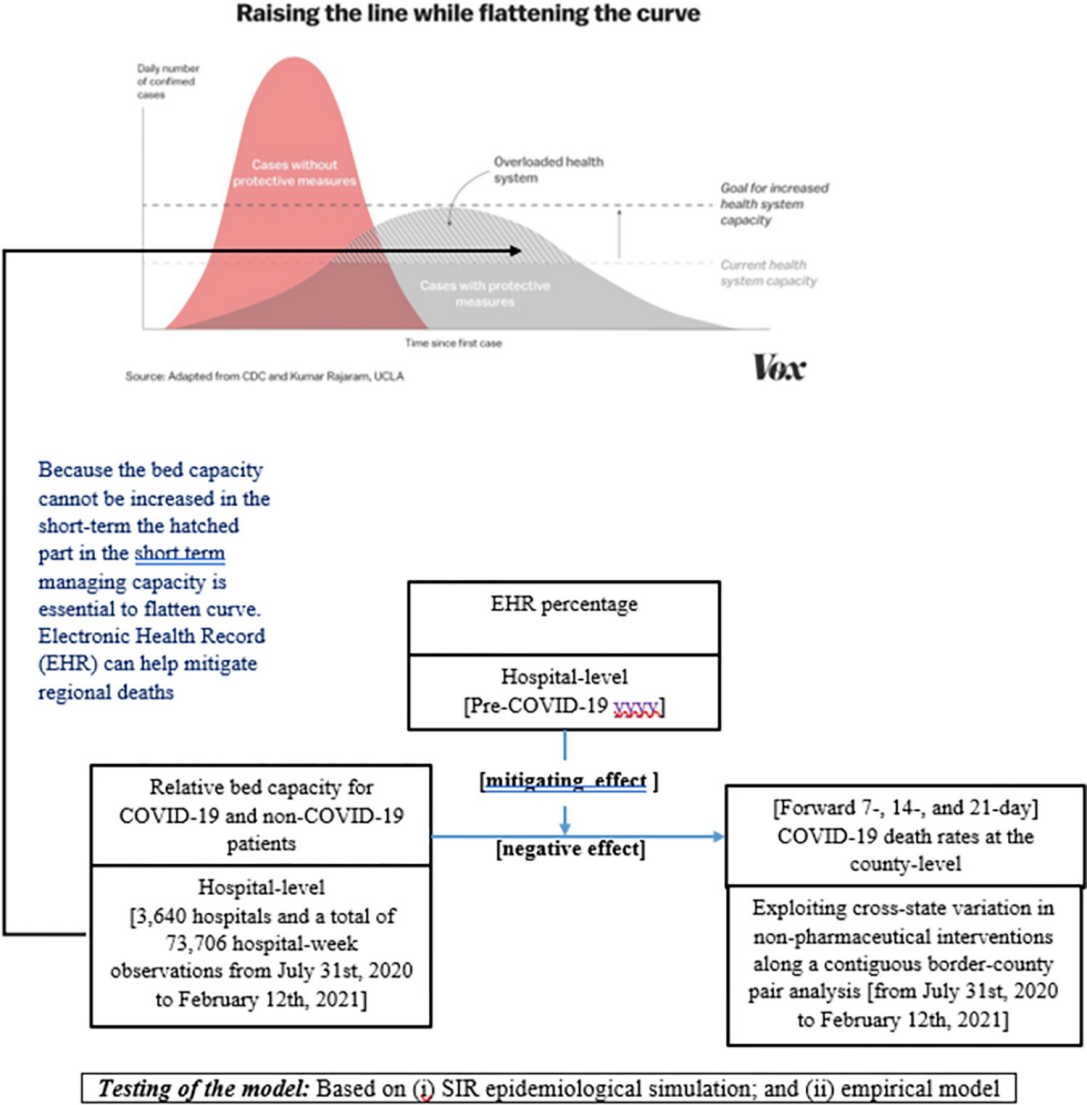
Conceptual model.

Our conceptual framework as illustrated in Fig 1, with sticky bed capacity, allocation of beds for COVID-19 and non-COVID-19 patients became a critical trade-off for hospitals [7, 9]. To overcome the sticky short-term bed capacity (i.e., to manage the hatched area in Fig 1), EHR systems could improve coordination across patient types and also strengthen local coordination in the healthcare operations ecosystem. We focused on the extent of pre-COVID-19 EHR adoption—both in health registries and technology—to predict whether such investments ameliorated the forward-looking local death rates of COVID-19 [18–20]. We use death rates after 7 days, 14 days and at 21 days (the periods when the probability of death was the highest after infection).

Elaborating on real and observed mechanisms, COVID-19 mortality outcomes are driven by a complex interchange and coordination of patient care needs in the local health ecosystem. As such, hospital-level outcomes of COVID-19 patients would censor the more intensive local coordination witnessed throughout the US during the pandemic. Pre-COVID-19 EHR adoption could help assess the impact on broader outcomes in the local health ecosystem by facilitating the flow of information, capacity, speed of response, and coordinated response necessary to lower COVID-19 mortality. Due to the variations in state-level non-pharmaceutical interventions [11, 21, 22] and patient-level heterogeneity in political ideologies, patients may also vary in the need and desire to take precautions and seek care. These heterogeneities make patient-level outcomes less meaningful as the variance explained by hospital actions would also be confounded by local social and political factors.

The line of sight from EHR coordination among hospitals and providers and COVID-19 death outcomes is representative of complex adaptive systems in the face of the pandemic [23]. Based on the critical realism perspective [24–26], managing the dual care needs of COVID-19 and non-COVID-19 patients represents the “real” world where microdynamics of patient care, potential confounds in spillovers between the two patient types, and a complex healthcare supply chain of interactions among stakeholders presents a challenging setting of the “observed” world providing perspective and experiences through the released data from the government. Consistent with critical realism, though we cannot observe the flows and stocks of coordination abilities in a local healthcare supply chain during a pandemic, it will be a possible epistemic fallacy to assume that we can ontologically test for the mechanisms driving individual patient level COVID-19 outcomes in the high-pressure, high-stakes pandemic context.

We also test for cross-state variation in non-pharmaceutical interventions along a contiguous border-county pair analysis, use 2SLS analysis based on quality ratings, and use foot-traffic data at the US hospitals COVID-19 death outcomes. Our study authors approached two practitioners in the healthcare field: 1) a cardiologist who is an advanced heart failure specialist; and 2) an oncologist who was also a hospitalist and worked during the peak of COVID-19 pandemic. We enquired about the bed capacity during the pandemic and how hospitals with variable EHR capabilities managed the patient flow with local hospitals. The practitioners stated that EHR systems helped with the bed allotment during the pandemic. In some hospitals with higher EHR capabilities, there were centralized bed allotment system that were connected to other nearby hospitals. Both practitioners agreed that our research question is valid and relevant to test.

To somewhat address the potential limitations of focusing on forward-looking local level death rates in a county, we use both a simulation based on the SIR model (provided in S1 File) and the available weekly empirical data. Our proposed model aims to make the following contributions. First, with relation to the Hippocratic oath of ‘do no harm,’ operational flexibility reflected in relative capacity of beds for COVID-19 and non-COVID-19 goals remained a critical challenge to lowering COVID-19 death rates [3–5]. In other words, a greater focus on testing, admitting, and managing both patient types must have played a critical role in lowering death rates under stickier short-term hospital bed capacity. With the US facing among the highest documented COVID-19 deaths in the world, hospital critical care is a continuing choke point, adding to increased stress points and fractures at multiple interfaces of hospital supply chains.

Though the demand for beds increased rapidly during COVID-19, hospitals also cut down on non-urgent care and postponed non-essential surgeries to make room for COVID-19 patients. At the core of their ability to manage capacity could be the extent of pre-COVID-19 EHR adoption. With strained bed capacity and intermittent surges, we expect that pre-COVID-19 EHR investments will help mitigate, but not alleviate forward-looking death rates. EHR adoption levels could play an elemental role in providing infrastructure for a spectrum of care needs at multiple levels of a local healthcare ecosystem. Though we do not imply that scaling EHR in the short term is possible, the findings add to the calls by experts [8, 27, 28] on the need for improved EHR adoption for future pandemics.

Second, from an operations management perspective, available bed capacity has been a critical constraint for hospitals during COVID-19 [27, 28]. With many hospitals running at capacity, managing the allocation of beds for COVID-19 patients remains a critical challenge. With unexpected case surges, coupled with the limited ability to scale capacity hospitals, it became equally critical to service COVID-19 patients while also ensuring those with non-COVID-19 co-morbidities received critical care. The relative capacity for COVID-19 and non-COVID-19 beds is a critical challenge to lowering future death rates in the local area. Our core premise is that EHR percentage levels from the pre-COVID-19 years could form the basis for managing capacity, coordinating with local hospitals and care providers, and thereby facilitating the improved death rates.

Third, empirically, we focused on a theory-based approach and drew inferences based on simulation and empirical analysis. Our is among the first study that leverages recently released weekly data on US hospitals and validates the propositions using a simulation and empirical study. Our empirical results are robust to a variety of specifications and also to contiguous county border-pair analysis controlling for spatial correlation varying between state variations in non-pharmaceutical intervention policies [29]. We also find support for the effects in a 2SLS model. We aim to leverage the weekly variation in bed allocations and forward-looking decline in death rates.

In the next section, we present a theoretical background, followed by the empirics. We have included simulation models in the S1 File. We then discuss the results, provide theoretical and practical implications and highlight limitations and future research directions. We list our conclusions in end.

## Theoretical background

Based on organizational information processing theory (OIPT) in the supply chain literature [30–32], during COVID-19 EHRs may be essential to managing uncertainty and complexity in the scale, uncertainty, and ambiguity of healthcare operations where bed capacity could not be expanded in the short-run [8, 10, 15]. EHR improves cost-savings, increases operational efficiency, enhances patient access and satisfaction, and most importantly, improves the overall quality of care [33–37]. EHR systems include digitization of physicians, lab, pharmacy, and nursing-related tasks, data, reports, and orders. EHR systems also provide clinical decision-making guidelines, reminders, and alerts, and facilitate the access, viewing of records and tests. EHR applications are multicontextual and multidimensional ranging from computerized orders to diagnostic decision-making and from vertical and lateral information exchange interfaces to coordinated response protocols. The studies have demonstrated the value of EHR in driving the cost logic, the coordination logic, and the decentralization logic to improve healthcare delivery, and clinical and administrative outcomes [38]. EHR helps administrative workers coordinate and manage information flows, and assists frontline workers in making complex and time-sensitive health delivery decisions.

Because the percentage of EHR adoption cannot also be adjusted in the short run during a pandemic, we focus on the pre-COVID-19 level EHR adoption in a hospital. In the past decade, the US government has provided significant incentives to invest in EHR systems. Among the most important federal law are the Health Information Technology for Economic and Clinical Health (HITECH) Act of 2009 and the Federal Health IT Strategic Plan [39]. As a part of the American Recovery and Reinvestment Act (ARRA), about $30 billion in incentives were provided for EHR adoption. Among the non-federal hospitals, EHR adoption was 84% in 2015 [40].

### EHR systems, local healthcare supply chains, and COVID-19 response

According to organizational information processing theory (OIPT) when facing complex, interdependent and uncertain tasks, firms must collate, analyze and use information [30–32]. COVID-19 significantly increased uncertainty, or the “difference between the amount of information required to perform the task and the amount of information already possessed by the organization” (Galbraith, 1973, p. 5) [41]. EHR may strengthen information processing capacity in an increasingly uncertain COVID-19 environment. Consistent with prior supply chain research applying OIPT [31, 42, 43] we consider OIPT as a critical lens in studying the beneficial effects of EHR in improving the relative COVID-19 and non-COVID-19 capacity allocations [17]. Based on OIPT, EHR allows for horizontal (among hospitals and care providers) and vertical (among labs and pharmacies) relationships through improved contact intensity, greater liaison, and stronger integration necessary to improving COVID-19 related response [44]. Vertical information processing helps improve the intake and processing of patients and the provision of necessary health inputs and post-discharge care. Similarly, horizontal information processing among hospitals improves data access, operational planning, and flexibility in managing the unpredictable and fluid COVID-19 environment.

Consistent with the supply chain literature, coordination among healthcare providers through EHR is essential to promoting the necessary coordination in a local decentralized healthcare ecosystem [45, 46]. Healthcare systems are “a decentralized supply chain largely characterized by a lack of coordination mechanisms (financial or contractual) among physicians, hospitals, and patients (i.e., Schmoltzi & Wallenburg, 2012; Tachizawa & Wong, 2015)” [47 p. 38] where EHR could be the necessary infrastructure to improve coordination. EHR data are essential to not only sharing and reusing the data to facilitate decision making but are also essential to supporting critical treatment and capacity sharing [48]. The collaborative data structures driven by the interoperability of EHR improve collaboration and are increasingly at the center stage of local response to COVID-19 [49–51]. EHR may drive responsive and resilient point-of-care solutions. EHR levels may facilitate the necessary development, implementation, and flow of data sharing and improved coordination and communication to develop a local quasi-coordinated response during the times of COVID-19 [28]. The local orchestration of information, data, and patients across labs, hospitals, clinics, and physicians helps improve care for those without COVID-19 and thereby more effectively manage the allocation of COVID-19 and non-COVID-19 care.

Facilitating EHR provides the necessary mode of vertical and horizontal flow of information, decisions, and patients in hospital supply chains. Facilitating the need for intensive information exchange and coordination across supply members, EHR acts as the necessary means to improve the inter-and intra-hospital flow of patients, supplies, and information [52]. EHR may enhance the integration across outpatient/inpatient, medical, nursing, and other departments to improve information sharing and collaboration [52–55]. Internal integration is essential to managing the relative capacity of bed capacity by meeting the need for frequent, timely, and accurate information sharing in real-time [52, 54–57]. EHR may further improve the timeliness and quality of care by improving interactions with patients by improving communication and interchange of important patient health information and records within and across the local healthcare providers. The information interchange is essential to managing capacity while ensuring care during COVID-19 [48]. With evolving needs of the local healthcare ecosystem under COVID-19 hospitals must have access to and share EHR information to improve the effective flow of resources and information.

During COVID-19, the shared interdependencies are at the core of managing scarce resources [58–60]. As the demand for hospital beds increases, the need for improved management of non-COVID-19 patients increases, where shared inputs and interdependencies could help improve the care of non-COVID-19 by delaying non-urgent care. EHRs are easily shared across care providers, to decentralize available information, draw on expertise to manage admissions and discharges. EHR improves the clinical dimension (care provider and patient interactions) and operational dimension (tools and technologies) to manage capacity, reducing errors and improving the speed of responsiveness during COVID-19 [15].

The complex interdependencies among a variety of EHR systems may be central to improving the efficacy of bed allocations during COVID-19 in managing scale and complexity to manage county-level death rates. The lateral and vertical interdependencies and coordination facilitated by EHR systems may improve the efficacy of relative bed allocation on local COVID-19 outcomes [61–63]. Improved information processing capabilities that allow for the creation and delivery of healthcare services by coordinating vertical and horizontal inter-dependencies across local healthcare providers, labs, insurance, and medical experts.

Relative bed allocation between COVID-19 and non-COVID-19 patients is rooted in operational flexibility. In allocating bed capacity, EHR levels may allow for essential inter-and intra-functional collaboration and information sharing may help improve flexibility through real-time information sharing to improve flexibility, speed, and responsiveness. At lower EHR levels, flexibility and integrative information processing may not be feasible. As hospitals try to intake COVID-19 patients, EHR may help speed up, re-prioritize or re-route non-COVID-19 patients to delay or postpone surgeries. Limited EHR percentage may limit the necessary close and intensive coordination to manage close and intensive coordination necessary to develop plans to respond to uncertainty in supply and demand, ad-hoc resolution of care decisions, and improving flexibility in healthcare services by serving patients with available resources during a pandemic.

Our SIR simulation model (in S1 File) [denoted by the number of susceptible people, the number of infected, and the number of recovered persons] shows that a coordinated solution facilitated by EHR may lead to lower COVID-19 death rates.

## Empirical analysis

### Data and sample

All data sources used for this study were publicly available and anonymized. The data on relative bed allocations are from the weekly reports from the hospitals in the ‘COVID-19 Reported Patient Impact and Hospital Capacity by Facility database.’ We use the earliest to the latest available weekly data, from July 31st, 2020 to February 12th, 2021. Hospitals registered with the Center for Medicare & Medicaid Services (CMS) are the respondents in the data, and the data are released by the Department of Health and Human Services. Additional details on the data are available from https://healthdata.gov/Hospital/COVID-19-Reported-Patient-Impact-and-Hospital-Capa/anag-cw7u The weekly data are from Friday to Thursday, and provide information on bed allocations for COVID-19 and non-COVID-19 beds, along with information on staffed beds and relative capacity of ICU beds. The data also provides the level of coverage in reporting of beds, the information we use to control for the quality of the information provided by hospitals.

We collect additional hospital facility-level EHR data from CMS’ Medicare EHR incentive program for providers. This dataset provides information on meaningful use measures among hospitals that received incentive payments for adopted, implemented, upgraded, or demonstrated meaningful use of certified EHR technology. Additional details are available at https://www.cms.gov/Regulations-and-Guidance/Legislation/EHRIncentivePrograms The EHR data for eligible hospitals can be obtained here: https://www.cms.gov/Regulations-and-Guidance/Legislation/EHRIncentivePrograms/DataAndReportsWe obtain county-level COVID-19 death data from *USAFacts*, an organization that collects data released by CDC and state-/local- public health agencies. Additional details on the COVID-19 data are available from https://usafacts.org/visualizations/coronavirus-covid-19-spread-map/ We also obtain hospital characteristics from the Hospital General Information dataset released by CMS. Additional details on the hospital general information data is available here https://data.cms.gov/provider-data/dataset/xubh-q36u Further, selected hospital personnel data was obtained from provider of service files from CMS. Refer to provider of service files at https://data.cms.gov/provider-characteristics/hospitals-and-other-facilities/provider-of-services-file-hospital-non-hospital-facilities/data. We then merge the COVID-19 data with the hospital capacity and EHR data. Our final sample consists of 3,640 hospitals and a total of 73,706 hospital week observations.

### Empirical specification

We use a random-effects model with robust standard errors on the panel data to predict the impact of EHR and relative capacity on future local COVID-19 death rates. Due to the time-invariant measure of pre-COVID-19 EHR extent of usage, we use the random-effects model. Our empirical specification is as follows:

$$Y_{cw,t+7,t+14,ort+21}=\alpha_{1}{EHR}_{h}+{\alpha_{2}RC}_{\left(hw\right)}+{\alpha_{3}({EHR}_{h}\times RC}_{\left(hw\right)})+X+\lambda_{w}+{(State}_{\left(h\right)}\times \lambda_{w})+\varepsilon_{hw}$$ (1)

Where *c* is the county, *h* is the hospital, *t* is the date of the weekly hospital report, and *w* is the week of the year. *Y*_*cw*_ is the t+7, t+14, and t+21 day COVID-19 death rates in the county. The death rates are per ‘000 population. We take a log of death rates per ‘000 population.

*RC*_(*hw*)_ is the relative capacity of beds for COVID-19 and non-COVID-19 patients. The measure is computed by dividing the total number of adult patients hospitalized that are confirmed or suspected of COVID-19 in a given week by the total number of beds (7-day average) in the hospital during the week.

*EHR*_*h*_ is the extent of pre-COVID adoption of EHR in a hospital. The measure includes two types of EHR measures. Both measures are based on the factor score derived from principal component analysis. The first measure is the extent of EHR usage based on seven items. Each item is reported as a percentage in the CMS hospital-level data. The seven items are: (i) Computerized Provider Order Entry (CPOE); (ii) share of medication orders recorded using CPOE; (iii) share of patients who have electronic access to their health information and use it; (iv) share of patients who have electronic access to their health information and use it within 36 hours after information is available; (v) share of patients who were subsequently provided patient-specific education resources; (vi) share of transitions of care where medication reconciliation was performed; and (vii) share of transitions of care where health information of patients was exchanged electronically.

The second measure of the EHR registry is a dichotomous measure based on whether or not a hospital report results to the public health agency. The five items are whether the hospital is active in engagement with public health agency in submitting: (i) electronic laboratory results; (ii) immunization data; (iii) specialized registry; (iv) whether the hospital is in active engagement with public health agency to submit syndromic surveillance data; and (v) whether the hospital uses clinical decision support to improve performance on high priority health conditions. Our coefficient of interest is *α*_3_, or the estimate of the interaction between *EHR*_*h*_ and *RC*_(*hw*)_.

*X* is a vector of controls. The controls include the type of hospital—critical access or short-term hospital, hospital ownership type (Federal hospitals are the omitted category), whether or not the hospital provides emergency services, hospital staffing (physicians, registered nurses, licensed practical or vocational nurses). To control for the quality of weekly reporting, we also include a set of coverage variables, an indicator of the number of times a hospital reported a particular line item during the week of data collection. We also include the prior total number of emergency department (ED) visits during the past seven days as controls. Finally, we include whether or not a hospital is located in a metro area.

To control for week × state fixed effects, we include (*State*_(*h*)_×*λ*_*w*_) where *State*_(*h*)_ is the state where the hospital is located and *λ*_*w*_ is the week of the year dummy. We control for state-week-of-the-year fixed effects to account for the ongoing changes in the state (e.g., changes in lockdown policies and mobility).

Table 1 presents the summary statistics of all the variables used in this study.

**Table 1:** Summary statistics.

Variable	N	mean	sd
***Dependent variables***			
COVID-19 county death rate (t+7) days	64,730	0.5572	0.3328
COVID-19 county death rate (t+14) days	60,728	0.5682	0.3298
COVID-19 county death rate (t+21) days	57,507	0.5788	0.3273
***Independent variables and covariates***			
Relative capacity	73,706	0.1042	0.1005
EHR factor analysis percentage	73,706	0.0010	0.8424
EHR factor analysis registry	73,706	0.0143	0.7174
Hospital type—critical access hospitals	73,706	0.1680	0.3739
Hospital ownership—Government hospital district	73,706	0.0986	0.2982
Hospital ownership—Government local	73,706	0.0658	0.2480
Hospital ownership—Government state	73,706	0.0135	0.1154
Hospital ownership—Physician	73,706	0.0145	0.1197
Hospital ownership—proprietary	73,706	0.1703	0.3759
Hospital ownership—voluntary nonprofit church	73,706	0.0787	0.2693
Hospital ownership—voluntary nonprofit other	73,706	0.0847	0.2785
Hospital ownership—voluntary nonprofit private	73,706	0.4712	0.4992
Hospital provides emergency services	73,706	0.9494	0.2191
Log of the total number of full-time equivalent physicians	73,706	1.5314	1.6872
Log of the total number of full-time equivalent registered nurses	73,706	4.9364	1.5523
Log of the total number of full-time equivalent licensed practical or vocational nurses	73,706	2.2114	1.4282
Total beds seven-day coverage	73,706	6.8683	0.5915
All adult hospital beds seven-day coverage	73,706	6.7385	1.1029
All adult hospital inpatient beds seven-day coverage	73,706	6.7490	1.0702
Inpatient beds used seven-day coverage	73,706	6.8602	0.6220
All adult hospital inpatient beds occupied seven-day coverage	73,706	6.8099	0.8371
Total adult patients hospitalized confirmed and suspected of COVID-19 seven day coverage	73,706	6.8596	0.6277
Total adult patients hospitalized confirmed COVID-19 coverage	73,706	6.8428	0.7185
Total pediatric patients hospitalized confirmed and suspected of COVID-19 coverage	73,706	6.8530	0.6493
Total pediatric patients hospitalized confirmed COVID-19 coverage	73,706	6.8157	0.7755
Inpatient beds seven day coverage	73,706	6.8410	0.7287
Total ICU beds seven-day coverage	73,706	6.8362	0.7688
Total staffed adult ICU beds seven-day coverage	73,706	6.7304	1.1264
ICU beds used seven-day coverage	73,706	6.8433	0.7291
Staffed adult ICU bed occupancy seven-day coverage	73,706	6.7679	0.9978
Staffed ICU adult patients confirmed and suspected of COVID-19 seven day coverage	73,706	6.8122	0.8587
Staffed ICU adult patients confirmed COVID-19 seven day coverage	73,706	6.8080	0.8773
Total patients hospitalized and confirmed of influenza seven-day coverage	73,706	4.3521	3.3187
ICU patients confirmed influenza seven-day coverage	73,706	4.3507	3.3193
Total patients hospitalized and confirmed of influenza and COVID-19 seven-day coverage	73,706	4.3475	3.3202
Previous day total ED visits seven-day sum	73,706	640.3113	663.8941
Hospital is in metro area	73,706	0.8590	0.3480
Instrumental variable: Hospital overall rating	64,783	3.1215	1.1177

### Main analysis

Table 2 presents the results of our main analysis. Models 1, 2, and 3 present the estimates of the impact of EHR percentage on relative capacity on county COVID-19 death rates in t+7, t+14, and t+21 days, respectively. We find that EHR adoption and relative capacity for COVID-19 and non-COVID-19 beds is associated with lower county COVID-19 death rates.

**Table 2:** Main results.

	(1)	(2)	(3)
VARIABLES	COVID-19 county death rate (t+7) days	COVID-19 county death rate (t+14) days	COVID-19 county death rate (t+21) days
Relative capacity	0.211***	0.250***	0.289***
	(0.0119)	(0.0123)	(0.0127)
EHR percentage	0.0107***	0.0107***	0.0108***
	(0.00163)	(0.00166)	(0.00171)
EHR percentage × Relative capacity	-0.188***	-0.197***	-0.207***
	(0.0103)	(0.0106)	(0.0109)
EHR registry	0.00460**	0.00418**	0.00377*
	(0.00182)	(0.00187)	(0.00193)
EHR registry × Relative capacity	-0.0798***	-0.0797***	-0.0807***
	(0.0105)	(0.0109)	(0.0114)
Hospital type—critical access hospitals	-0.0323***	-0.0352***	-0.0361***
	(0.00442)	(0.00469)	(0.00490)
Hospital ownership—Government hospital district	0.187***	0.188***	0.189***
	(0.0114)	(0.0118)	(0.0120)
Hospital ownership—Government local	0.210***	0.212***	0.213***
	(0.0120)	(0.0125)	(0.0127)
Hospital ownership—Government state	0.139***	0.139***	0.139***
	(0.0130)	(0.0135)	(0.0137)
Hospital ownership—Physician	0.186***	0.189***	0.192***
	(0.0124)	(0.0128)	(0.0130)
Hospital ownership—proprietary	0.206***	0.206***	0.207***
	(0.0111)	(0.0115)	(0.0117)
Hospital ownership—voluntary nonprofit church	0.165***	0.165***	0.164***
	(0.0113)	(0.0117)	(0.0119)
Hospital ownership—voluntary nonprofit other	0.142***	0.142***	0.142***
	(0.0113)	(0.0117)	(0.0119)
Hospital ownership—voluntary nonprofit private	0.164***	0.165***	0.165***
	(0.0111)	(0.0115)	(0.0116)
Hospital provides emergency services	-0.0507***	-0.0520***	-0.0527***
	(0.00376)	(0.00386)	(0.00397)
Log of total number of full-time equivalent physicians	0.00706***	0.00700***	0.00689***
	(0.000584)	(0.000593)	(0.000604)
Log of total number of full-time equivalent registered nurses	0.00487***	0.00416***	0.00377***
	(0.000762)	(0.000762)	(0.000762)
Log of total number of full-time equivalent licensed practical or vocational nurses	0.00409***	0.00484***	0.00587***
	(0.000695)	(0.000710)	(0.000724)
Total beds seven day coverage	-0.000827	-0.000485	-0.00386
	(0.00357)	(0.00371)	(0.00361)
All adult hospital beds seven day coverage	0.0103***	0.0108***	0.0113***
	(0.00183)	(0.00191)	(0.00191)
All adult hospital inpatient beds seven day coverage	-0.00960***	-0.0102***	-0.0101***
	(0.00249)	(0.00258)	(0.00266)
Inpatient beds used seven day coverage	0.00826*	0.00877*	0.00915*
	(0.00470)	(0.00503)	(0.00506)
All adult hospital inpatient beds occupied seven day coverage	-0.00547**	-0.00501*	-0.00656**
	(0.00258)	(0.00271)	(0.00274)
Total adult patients hospitalized confirmed and suspected of COVID-19 seven day coverage	-0.0277***	-0.0261***	-0.0228***
	(0.00738)	(0.00775)	(0.00772)
Total adult patients hospitalized confirmed of COVID-19 coverage	0.0231***	0.0203***	0.0168***
	(0.00587)	(0.00616)	(0.00624)
Total pediatric patients hospitalized confirmed and suspected of COVID-19 coverage	0.00378	0.00327	0.00259
	(0.00558)	(0.00577)	(0.00579)
Total pediatric patients hospitalized confirmed of COVID-19 coverage	-4.84e-05	0.000970	0.00102
	(0.00422)	(0.00431)	(0.00422)
Inpatient beds seven day coverage	-0.00764*	-0.00813**	-0.00590
	(0.00396)	(0.00408)	(0.00423)
Total ICU beds seven day coverage	0.0109***	0.0112***	0.0107**
	(0.00377)	(0.00397)	(0.00423)
Total staffed adult ICU beds seven day coverage	-0.00571***	-0.00617***	-0.00664***
	(0.00221)	(0.00232)	(0.00240)
ICU beds used seven day coverage	-0.00883**	-0.0106**	-0.00950*
	(0.00439)	(0.00474)	(0.00491)
Staffed adult ICU bed occupancy seven day coverage	0.0119***	0.0141***	0.0139***
	(0.00276)	(0.00292)	(0.00297)
Staffed ICU adult patients confirmed and suspected of COVID-19 seven day coverage	0.0119*	0.0119*	0.0127*
	(0.00658)	(0.00706)	(0.00689)
Staffed ICU adult patients confirmed of COVID-19 seven day coverage	-0.00691	-0.00724	-0.00472
	(0.00636)	(0.00678)	(0.00665)
Total patients hospitalized and confirmed of influenza seven day coverage	0.0202***	0.0210***	0.0200***
	(0.00495)	(0.00484)	(0.00463)
ICU patients confirmed of influenza seven day coverage	-0.00566	-0.00659	-0.00664
	(0.00580)	(0.00573)	(0.00547)
Total patients hospitalized and confirmed of influenza and COVID-19 seven day coverage	-0.0206***	-0.0195***	-0.0178***
	(0.00356)	(0.00347)	(0.00331)
Previous day total ED visits seven day sum	3.61e-06	2.23e-06	1.14e-06
	(2.36e-06)	(2.16e-06)	(1.94e-06)
Hospital is in metro area	-0.0519***	-0.0563***	-0.0639***
	(0.00457)	(0.00486)	(0.00507)
Constant	0.386***	0.397***	0.404***
	(0.0177)	(0.0181)	(0.0187)
Week of the year fixed effects	Yes	Yes	Yes
Week of the year fixed effects × State fixed effects	Yes	Yes	Yes
Observations	64,728	60,724	57,504
R-squared	0.594	0.594	0.591

Robust standard errors in parenthesis

*** p<0.01

** p<0.05

* p<0.1

**Fig 2:**
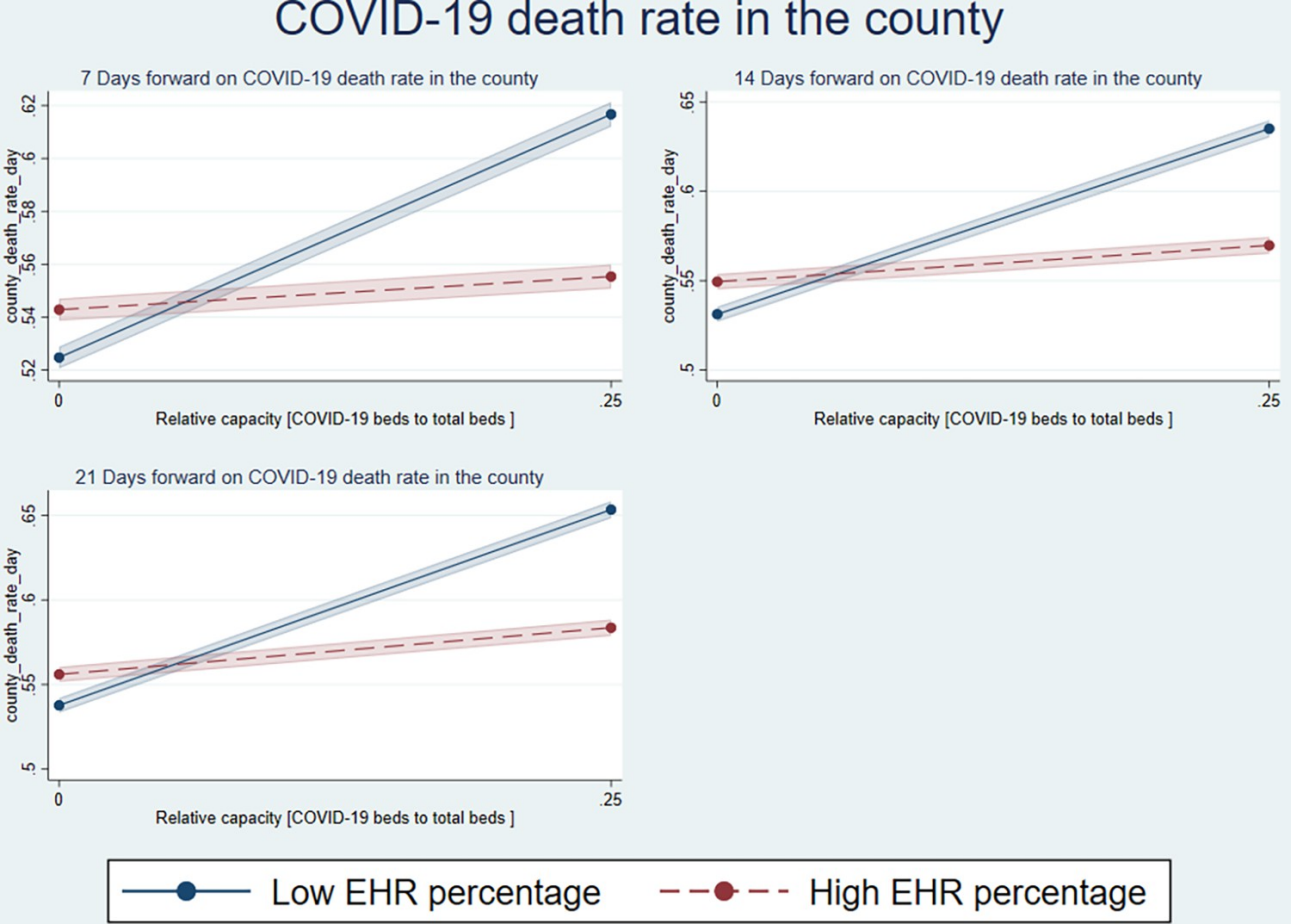
Relative capacity and EHR percentage on local COVID-19 death rate.

**Fig 3:**
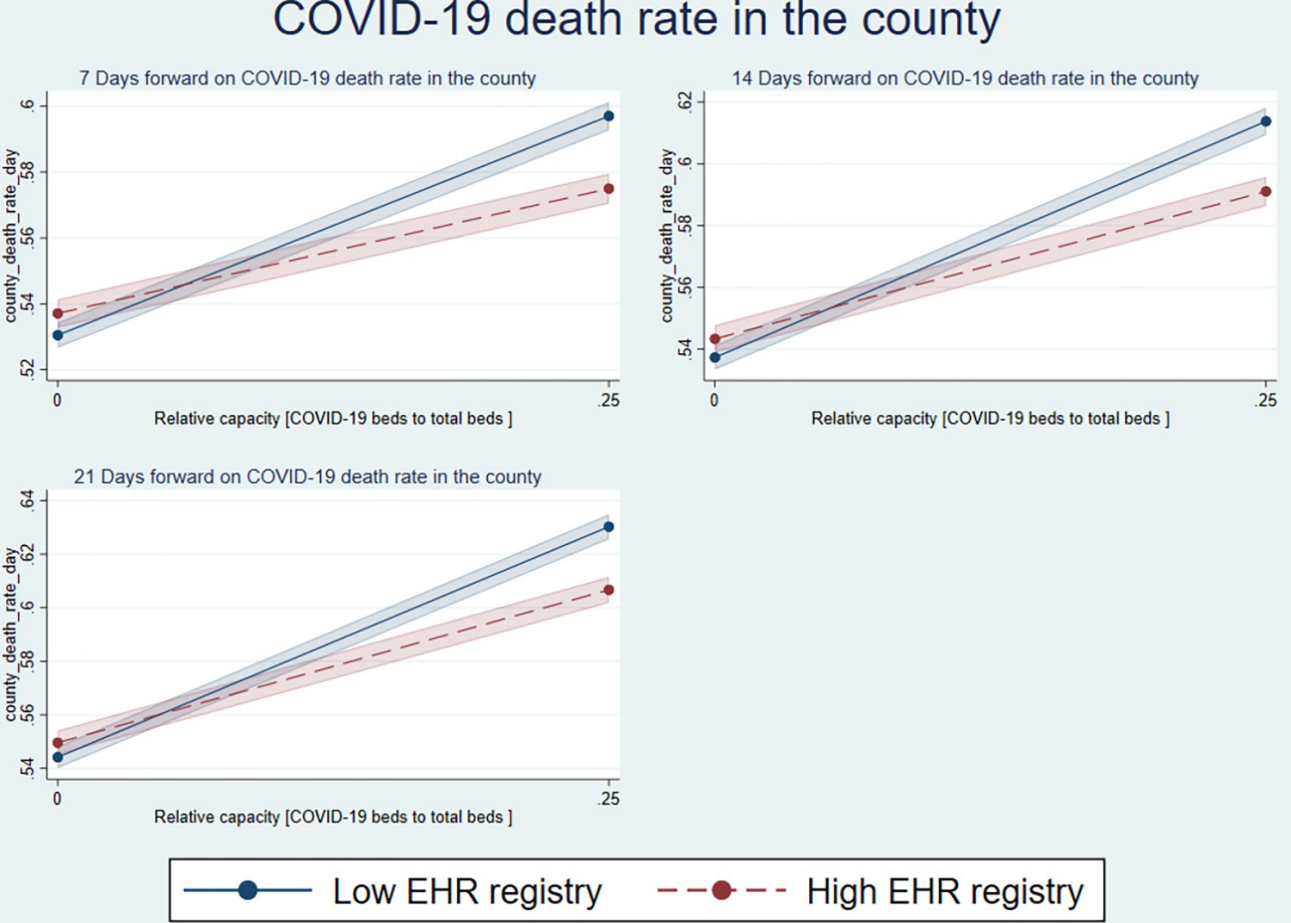
Relative capacity and EHR registry on local COVID-19 death rate.

Fig 2 shows that with the increasing relative capacity of beds for COVID-19 and non-COVID-19 beds, a higher EHR percentage was associated with a lower incidence of death rates (the dashed line). Similarly, Fig 3 shows similar inferences for higher EHR registry and death rates.

### Impact on foot traffic at hospitals

We also test whether the relative capacity of beds and EHR adoption impact foot traffic to hospitals after 14 days (the infection period at the time for the virus strain). Both the local COVID-19 incidence and death rates could impact the foot traffic at hospitals. We obtain daily foot traffic data from SafeGraph, a data company that compiles anonymized location data to various points of interest. We obtained the data for all the hospitals and health locations (NAICS sector 6221, 6222, 6223) and then match it with our study sample of hospitals. We then compute the cumulative foot traffic rate until time, t (defined as the natural log of cumulative foot traffic to the hospital until time, t per 1,000 county population + 1).

Our empirical specification is as follows:

$${Foot\;traffic}_{hw,t+14days}=\alpha_{1}{EHR}_{h}+{\alpha_{2}RC}_{\left(hw\right)}+{\alpha_{3}({EHR}_{h}\times RC}_{\left(hw\right)})+\alpha_{4}{Covid19Incid}_{cwt}+{\alpha_{5}({Covid19Incid}_{cwt}\times RC}_{\left(hw\right)})+{\alpha_{6}Covid19Death}_{cwt}+{\alpha_{7}({Covid19Death}_{cwt}\times RC}_{\left(hw\right)})+X+\lambda_{w}+{(State}_{\left(h\right)}\times \lambda_{w})+\varepsilon_{hw}$$ (2)

Where the outcome variable is the cumulative foot traffic rate at the hospitals until time t+14 days. *Covid19Incid* is the cumulative COVID-19 incidence rate at the county, c until time t in week w. *Covid19Death* is the cumulative COVID-19 death rate at the county, c until time, t in week w. We also include the interaction of EHR adoption (percentage and registry) with cumulative COVID-19 incidence rate (and death rate) until time, t. All other controls are the same as those in Eq (1).

Table 3 shows the results of the impact of EHR percentage (and EHR registry) and relative capacity on cumulative foot traffic rate at the hospitals after 14 days. Model 1 shows the results without controls but includes the fixed effects, and Model 2 includes all controls. We find that for our preferred model (Model 2), the interaction between relative capacity at the time, t, and EHR registry was negative and significant on the cumulative foot traffic rate after t+14 days. We also find that the interaction of EHR (percentage and registry) with cumulative county incidence rates were positively associated with foot traffic. Further, the cumulative county death rates interaction with EHR percentage was negatively related to foot traffic.

**Table 3:** Impact on foot traffic at hospitals.

	(1)	(2)
VARIABLES	Cumulative foot traffic rate to hospitals at t+14 days	Cumulative foot traffic rate to hospitals at t+14 days
Relative capacity at time, t	1.059***	0.939***
	(0.0768)	(0.0711)
EHR percentage	-0.0796***	-0.0457
	(0.0305)	(0.0302)
EHR percentage × Relative capacity	-0.410***	-0.0882
	(0.0774)	(0.0710)
EHR registry	-0.161***	-0.118***
	(0.0393)	(0.0356)
EHR registry × Relative capacity	-0.650***	-0.307***
	(0.0820)	(0.0707)
Cumulative county incidence rate of COVID-19 at time, t	-0.626***	-0.378***
	(0.0174)	(0.0165)
Cumulative county death rate of COVID-19 at time, t	-0.0593*	-0.280***
	(0.0319)	(0.0297)
EHR percentage × Cumulative county incidence rate of COVID-19	0.0350***	0.0194*
	(0.0120)	(0.0116)
EHR percentage × Cumulative county death rate of COVID-19	-0.113***	-0.136***
	(0.0281)	(0.0265)
EHR registry × Cumulative county incidence rate of COVID-19	0.0566***	0.0255*
	(0.0157)	(0.0138)
EHR registry × Cumulative county death rate of COVID-19	-0.0386	-0.0247
	(0.0379)	(0.0334)
Constant	5.728***	5.490***
	(0.0503)	(0.142)
Week of the year fixed effects	Yes	Yes
Week of the year fixed effects × State fixed effects	Yes	Yes
All additional controls as in Table 2	No	Yes
Observations	56,154	54,649
R-squared	0.426	0.499

Robust standard errors in parenthesis

*** p<0.01, ** p<0.05, * p<0.1

### Analysis by components of EHR

Table 4 presents the results of interactions by each component of EHR items used for the principal components analysis used to compute EHR percentage and EHR registry measures. We find that higher relative capacity was associated with lower local COVID-19 death rates when the facility had a higher share of medication orders recorded using Computerized Provider Order Entry; share of patients having electronic access to their health information, and used it or shared it for transitions of care where medication reconciliation/electronic exchange of health information was performed.

**Table 4:** Analysis by EHR components.

	(1)	(2)	(3)
VARIABLES	COVID-19 county death rate (t+7) days	COVID-19 county death rate (t+14) days	COVID-19 county death rate (t+21) days
Share of laboratory orders recorded using Computerized Provider Order Entry (CPOE)	0.105***	0.104***	0.105***
	(0.0107)	(0.0110)	(0.0113)
Share of medication orders recorded using Computerized Provider Order Entry (CPOE)	-0.0113	-0.00395	4.66e-05
	(0.0162)	(0.0167)	(0.0172)
Share of patients who have electronic access to their health information and use it	-0.0617***	-0.0713***	-0.0718***
	(0.0105)	(0.0110)	(0.0113)
Share of patients who have electronic access to their health information and use it within 36 hours after information is available	-0.186***	-0.192***	-0.195***
	(0.0121)	(0.0125)	(0.0129)
Share of patients who were subsequently provided patient-specific education resources	0.0412***	0.0439***	0.0405***
	(0.00620)	(0.00640)	(0.00657)
Share of transitions of care where medication reconciliation was performed	0.0471***	0.0548***	0.0548***
	(0.0127)	(0.0131)	(0.0134)
Share of transitions of care where heath information of patients were exchanged electronically	0.0426***	0.0414***	0.0449***
	(0.00609)	(0.00626)	(0.00645)
Hospital is in active engagement with public health agency to submit electronic laboratory results	-0.0854***	-0.0914***	-0.0999***
	(0.00853)	(0.00873)	(0.00905)
Hospital is in active engagement with public health agency to submit immunization data	-0.0182	-0.0185	-0.0164
	(0.0125)	(0.0128)	(0.0131)
Hospital is in active engagement to submit data to specialized registry	-0.0341***	-0.0357***	-0.0374***
	(0.00381)	(0.00393)	(0.00405)
Hospital is in active engagement with public health agency to submit syndromic surveillance data	-0.00598	-0.00816	-0.00935*
	(0.00521)	(0.00538)	(0.00556)
Relative capacity	1.558***	1.523***	1.493***
	(0.195)	(0.199)	(0.206)
Share of laboratory orders recorded using Computerized Provider Order Entry (CPOE) × Relative capacity	-0.524***	-0.550***	-0.593***
	(0.0738)	(0.0756)	(0.0782)
Share of medication orders recorded using Computerized Provider Order Entry (CPOE) × Relative capacity	-0.421***	-0.517***	-0.601***
	(0.107)	(0.110)	(0.113)
Share of patients who have electronic access to their health information and use it × Relative capacity	-0.451***	-0.400***	-0.394***
	(0.0784)	(0.0813)	(0.0836)
Share of patients who have electronic access to their health information and use it within 36 hours after information is available × Relative capacity	0.0513	0.108	0.130
	(0.0803)	(0.0831)	(0.0869)
Share of patients who were subsequently provided patient-specific education resources × Relative capacity	0.105**	0.0988**	0.123***
	(0.0409)	(0.0423)	(0.0440)
Share of transitions of care where medication reconciliation was performed × Relative capacity	-0.466***	-0.514***	-0.512***
	(0.0786)	(0.0813)	(0.0843)
Share of transitions of care where heath information of patients were exchanged electronically × Relative capacity	-0.131***	-0.121***	-0.138***
	(0.0402)	(0.0410)	(0.0422)
Hospital is in active engagement with public health agency to submit electronic laboratory results × Relative capacity	0.0926	0.148**	0.209***
	(0.0579)	(0.0585)	(0.0596)
Hospital is in active engagement with public health agency to submit immunization data × Relative capacity	-0.0750	-0.0596	-0.0718
	(0.0737)	(0.0762)	(0.0791)
Hospital is in active engagement to submit data to specialized registry × Relative capacity	0.0976***	0.110***	0.129***
	(0.0262)	(0.0269)	(0.0279)
Hospital is in active engagement with public health agency to submit syndromic surveillance data × Relative capacity	-0.169***	-0.164***	-0.154***
	(0.0346)	(0.0359)	(0.0378)
Constant	0.644***	0.668***	0.690***
	(0.0368)	(0.0374)	(0.0387)
All controls	Included	Included	Included
Week of the year fixed effects	Yes	Yes	Yes
Week of the year fixed effects × State fixed effects	Yes	Yes	Yes
Observations	64,728	60,724	57,504
R-squared	0.602	0.602	0.600

Robust standard errors in parenthesis

*** p<0.01

** p<0.05

* p<0.1

### Analysis by quartile of county death rate

The COVID-19 caseload varied significantly by region and city in the US. To assess whether our findings are an artifact of effects-driven by counties that experienced lower COVID-19 caseloads, and therefore, demonstrating greater efficacy of pre-COVID-19 EHR efficacy, we conduct a quantile regression analysis. We split the sample by quartiles of death rates in each county. Table 5 presents the results for each quartile of a county death rate. We find that our main results are consistent across different quartiles of local death rates, especially for EHR percentage and relative capacity interaction.

**Table 5:** Analysis by quartile of county death rate.

	Quartile of county death rate	Relative capacity	EHR factor analysis percentage	EHR percentage × Relative capacity	EHR factor analysis registry	EHR registry × Relative capacity
COVID-19 county death rate (t+7) days	1	0.152***	0.00164	-0.0575***	0.00723***	-0.0421***
		(0.0104)	(0.00119)	(0.00812)	(0.00131)	(0.00864)
	2	0.0643***	-0.000850	-0.0157***	-0.00384***	0.0151**
		(0.00652)	(0.000813)	(0.00536)	(0.000979)	(0.00603)
	3	0.0380***	-0.00177**	-0.00143	-0.000230	-0.000404
		(0.00610)	(0.000831)	(0.00499)	(0.000866)	(0.00452)
	4	-0.0454**	0.0148***	-0.0723***	0.0195***	-0.0160
		(0.0195)	(0.00241)	(0.0165)	(0.00333)	(0.0209)
COVID-19 county death rate (t+14) days	1	0.192***	0.000206	-0.0620***	0.00554***	-0.0412***
		(0.0121)	(0.00134)	(0.00933)	(0.00144)	(0.00950)
	2	0.107***	-0.00131	-0.0221***	-0.00567***	0.0216***
		(0.00792)	(0.000939)	(0.00635)	(0.00112)	(0.00694)
	3	0.0802***	-0.00173*	-0.00795	-0.000701	-0.00478
		(0.00767)	(0.000935)	(0.00596)	(0.000956)	(0.00560)
	4	-0.0154	0.0152***	-0.0734***	0.0193***	-0.0220
		(0.0203)	(0.00245)	(0.0169)	(0.00341)	(0.0215)
COVID-19 county death rate (t+21) days	1	0.231***	-6.91e-05	-0.0708***	0.00525***	-0.0441***
		(0.0144)	(0.00155)	(0.0111)	(0.00159)	(0.0106)
	2	0.155***	-0.00177	-0.0249***	-0.00673***	0.0233***
		(0.00974)	(0.00108)	(0.00765)	(0.00126)	(0.00868)
	3	0.121***	-0.00167	-0.0187**	-0.00204*	-0.00492
		(0.00934)	(0.00107)	(0.00735)	(0.00107)	(0.00669)
	4	0.0214	0.0174***	-0.0918***	0.0191***	-0.0337
		(0.0209)	(0.00251)	(0.0174)	(0.00354)	(0.0219)

All controls are included in all models. The results presented in each row are obtained from regressions for each quartile. Robust standard errors in parenthesis

*** p<0.01

** p<0.05

* p<0.1

### Analysis by hospital type

We further analyze whether there is heterogeneity in results by type of hospitals–namely, critical access hospitals and short-term hospitals. In Table 6, we find that our main findings are consistent for short-term and critical access hospitals for EHR percentage interaction. We find consistent results for EHR registry interaction for short term hospitals.

**Table 6:** Analysis by hospital type.

Critical Access Hospitals	(1)	(2)	(3)
VARIABLES	COVID-19 county death rate (t+7) days	COVID-19 county death rate (t+14) days	COVID-19 county death rate (t+21) days
Relative capacity	0.170***	0.199***	0.205***
	(0.0357)	(0.0381)	(0.0399)
EHR percentage	0.0171***	0.0167***	0.0215***
	(0.00471)	(0.00500)	(0.00532)
EHR percentage × Relative capacity	-0.0749*	-0.0916**	-0.142***
	(0.0398)	(0.0427)	(0.0457)
EHR registry	-0.0192***	-0.0242***	-0.0283***
	(0.00505)	(0.00543)	(0.00580)
EHR registry × Relative capacity	0.0533	0.0809*	0.0658
	(0.0390)	(0.0415)	(0.0435)
Constant	0.498***	0.482***	0.502***
	(0.0359)	(0.0384)	(0.0393)
All controls	Included	Included	Included
Week of the year fixed effects	Yes	Yes	Yes
Week of the year fixed effects × State fixed effects	Yes	Yes	Yes
Observations	8,603	7,446	6,750
R-squared	0.686	0.695	0.697
Short Term Hospitals	(4)	(5)	(6)
VARIABLES	COVID-19 county death rate (t+7) days	COVID-19 county death rate (t+14) days	COVID-19 county death rate (t+21) days
Relative capacity	0.256***	0.299***	0.343***
	(0.0125)	(0.0129)	(0.0133)
EHR percentage	0.00543***	0.00589***	0.00500***
	(0.00169)	(0.00173)	(0.00176)
EHR percentage × Relative capacity	-0.167***	-0.177***	-0.180***
	(0.0105)	(0.0108)	(0.0112)
EHR registry	0.0101***	0.00982***	0.00959***
	(0.00187)	(0.00191)	(0.00195)
EHR registry × Relative capacity	-0.0930***	-0.0954***	-0.0950***
	(0.0108)	(0.0112)	(0.0117)
Constant	0.414***	0.435***	0.432***
	(0.0213)	(0.0218)	(0.0225)
All controls	Included	Included	Included
Week of the year fixed effects	Yes	Yes	Yes
Week of the year fixed effects × State fixed effects	Yes	Yes	Yes
Observations	56,001	53,147	50,624
R-squared	0.595	0.593	0.591

Robust standard errors in parenthesis

*** p<0.01

** p<0.05

* p<0.1

### Contiguous border county-pair analysis

**Fig 4:**
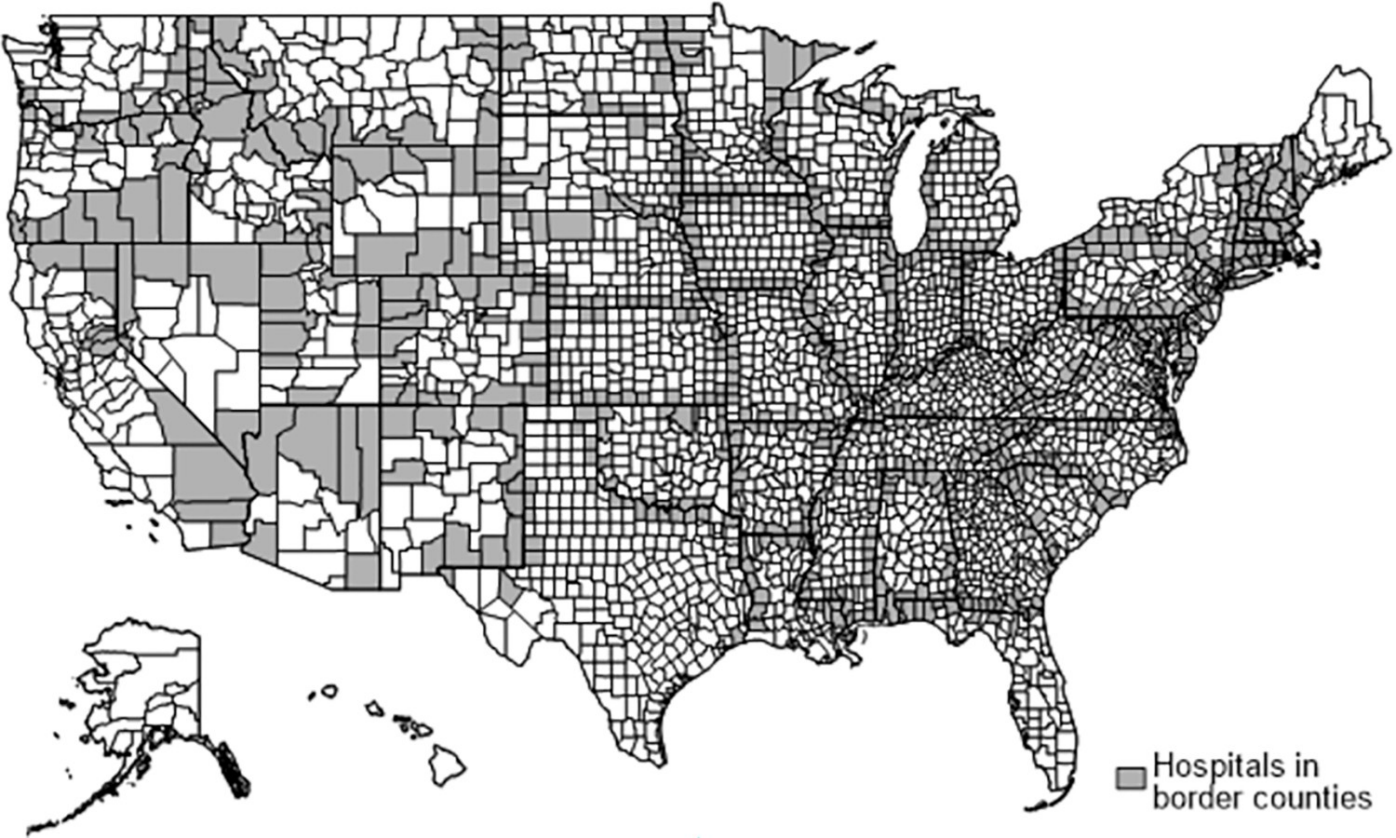
Border county analysis. *Note*. Reprinted from–maptile- package output maps in Stata 17 under a Unlicense license, a free and unencumbered software released into the public domain. For the full legal text of the Unlicense, see http://unlicense.org, original copyright 2023.

As a robustness test, we also perform a spatial heterogeneity analysis by comparing hospitals between counties bordering contiguous states. This design allows us to control for unobserved spatial factors that could be correlated with outcomes and considers potential spillover effects across state borders between adjacent counties from states with different COVID-19 policies. Fig 4 shows the border counties that contain at least one hospital and are used in the analysis. Our specification is as follows:

$$Y_{cw,t+7,t+14,or\;t+21}=\alpha_{1}{EHR}_{h}+{\alpha_{2}RC}_{\left(hw\right)}+{\alpha_{3}({EHR}_{h}\times RC}_{\left(hw\right)})+X+\lambda_{w}+{(County\;pair}_{\left(hcm\right)}\times \lambda_{w})+\varepsilon_{hcmw}$$ (3)

Where *h* is the hospital, *c* is the county, *m* is the adjacent county across the state border, and *w* is the week. *X* is a set of controls as described in Eq (1). We include the interaction of week of the year dummy and (*λ*_*w*_) and county pair dummy.

Table 7 shows the results of our analysis. We find that our original findings of higher relative capacity with higher EHR percentage or registry are associated with both lower county death rates at 7-, 14-, and 21-day forward-looking periods.

**Table 7:** Border county analysis.

	(1)	(2)	(3)
VARIABLES	COVID-19 county death rate (t+7) days	COVID-19 county death rate (t+14) days	COVID-19 county death rate (t+21) days
Relative capacity	0.0533***	0.0550***	0.0602***
	(0.0146)	(0.0156)	(0.0157)
EHR percentage	0.00532***	0.00507***	0.00536***
	(0.00155)	(0.00155)	(0.00158)
EHR percentage × Relative capacity	-0.0346***	-0.0341***	-0.0380***
	(0.00851)	(0.00869)	(0.00890)
EHR registry	0.0230***	0.0217***	0.0208***
	(0.00204)	(0.00206)	(0.00206)
EHR registry × Relative capacity	-0.0578***	-0.0537***	-0.0509***
	(0.00901)	(0.00918)	(0.00934)
Constant	0.658***	0.697***	0.711***
	(0.0400)	(0.0390)	(0.0357)
All controls	Included	Included	Included
Week of the year fixed effects	Yes	Yes	Yes
Week of the year fixed effects × State fixed effects	Yes	Yes	Yes
Observations	17,804	16,741	15,856
R-squared	0.942	0.942	0.942

Robust standard errors in parenthesis

*** p<0.01

** p<0.05

* p<0.1

### Endogeneity tests

Though the contiguous county pair analysis across state borders allows control for spatial spillovers from neighboring states, endogeneity is also a concern. We use overall hospital quality star rating as an instrument to predict relative capacity. The star rating is released in the year 2020 is derived from hospitals that report through Inpatient/Outpatient Quality Reporting program. See CMS link on overall hospital quality star ratings here: https://data.cms.gov/provider-data/topics/hospitals/overall-hospital-quality-star-rating and the data can be obtained from https://data.cms.gov/provider-data/dataset/xubh-q36uThe rating takes the weighted average scores of seven groups of measures such as mortality, the safety of care, readmission, patient experience, the effectiveness of care, timeliness of care, and efficient use of medical imaging. These ratings are based on the information before the onset of COVID-19.

We expect the direction of effect in the first stage of 2SLS to be negative for the following reasons. A hospital with a lower rating may be overwhelmed with the flow of COVID-19 patients and they may over-allocate beds to COVID-19 patients and significantly delay care of non-COVID-19 patients. A hospital with a higher rating may not be that quickly overwhelmed, it would have the capacity to set additional beds and scale of rising demand without significantly lowering care for non-COVID-19 patients. The star rating may have a direct influence on relative capacity as higher quality hospitals would have resources and infrastructure to allocate beds to COVID-19 patients and also more effectively manage the care of non-COVID-19 patients during a pandemic.

Our first stage results are shown in Table 8 Model 1. We find that the overall star rating of hospitals has a reduced effect on the relative capacity of COVID-19 patients concerning non-COVID-19 patients.

We then predict the relative capacity and use them in our second stage equation as follows:

$$Y_{cw,t+7,t+14,or\;t+21}=\alpha_{1}{EHR}_{h}+{\alpha_{2}Predicted\;RC}_{\left(hw\right)}+{\alpha_{3}({EHR}_{h}\times Predicted\;RC}_{\left(hw\right)})+X+\lambda_{w}+{(State}_{\left(h\right)}\times \lambda_{w})+\varepsilon_{hw}$$ (4)

The second stage results are presented in Models 2 to 4 of Table 8. Consistent with our main results, we find that higher predicted relative capacity and higher EHR adoption reduces county death rates.

**Table 8:** Two-stage least squares estimates.

	(1)	(2)	(3)	(4)
	First stage	Second Stage		
VARIABLES	Relative capacity	COVID-19 county death rate (t+7) days	COVID-19 county death rate (t+14) days	COVID-19 county death rate (t+21) days
Instrument: Hospital overall star rating	-0.00238***			
	(0.000281)			
Predicted Relative capacity		9.370***	9.401***	9.460***
		(0.354)	(0.362)	(0.371)
EHR factor analysis percentage	0.00229***	0.0943***	0.0946***	0.0885***
	(0.000386)	(0.0117)	(0.0122)	(0.0126)
EHR percentage × Predicted relative capacity		-1.078***	-1.091***	-1.050***
		(0.0995)	(0.104)	(0.108)
EHR factor analysis registry	0.00127**	0.0202	0.0299**	0.0215
	(0.000548)	(0.0143)	(0.0151)	(0.0159)
EHR registry × Predicted relative capacity		-0.291**	-0.380***	-0.309**
		(0.123)	(0.130)	(0.137)
Constant	0.155***	-0.996***	-0.982***	-0.994***
	(0.00819)	(0.0571)	(0.0586)	(0.0600)
All controls	Included	Included	Included	Included
Week of the year fixed effects	Yes	Yes	Yes	Yes
Week of the year fixed effects × State fixed effects	Yes	Yes	Yes	Yes
Cragg-Donald Wald F statistic		22	17.36	13.36
Anderson-Rubin Wald test		99.56	97.68	95.58
Observations	64,780	57,870	54,534	51,763
R-squared	0.476	0.599	0.595	0.591

Robust standard errors in parenthesis

*** p<0.01

** p<0.05

* p<0.1

### Discussion

#### Summary of findings

During COVID-19, the EHR could have improved the ability to manage care through the relative capacity of beds could have been a critical element improving COVID-19 death rates. Based on both a simulation and empirical exercise using pre-COVID-19 EHR penetration and weekly allocation of COVID-19 vs. non-COVID-19 beds, we find that local incidence of death rates decline. With sticky hospital bed capacity, EHR percentage is associated with meaningful impact on lower case and death rate at 7-, 14-, and 21-days in the future. The findings provide a simulation-based and empirical test on the extent of EHR presence in improving the hospital bed allocation and therefore death rates. Our findings are robust to a variety of specifications, contiguous border-county pairs, and 2SLS estimates.

A significant body of healthcare supply chain literature has focused on the value of EHR [64]. As local health care ecosystems struggle to respond to the pandemic, based on the swift-even flow framework [64], the relative capacity of beds and leveraging EHR may be important to managing operational flexibility [65]. Though operational efficiency is not the end goal in a pandemic, EHR may be necessary plumbing to not only provide the necessary hardware for information interchange, but in times of a pandemic, may provide the necessary interfaces to improve the relational capital through improved trust, knowledge, and information exchange [66].

The study responds to the growing calls for studying the implications of the COVID-19 pandemic for the hospital supply chain. As one of the early studies on the impact of EHR on local death rates, the empirical design allows for the test for the efficacy of EHR in times of crisis. The shock of the pandemic was unexpected and it, therefore, allows for a quasi-experimental design in a supply chain that is not only tightly regulated but the one that has faced significant pressures, fissures, and disconnects. The findings also contribute to the larger literature on supply chain resilience [67, 68] and responsiveness [69]. Vertical and lateral interdependencies managed by EHR in the local pandemic milieu are an important consideration. This study, therefore, aims to address the dearth of understanding on how hospitals managed bed capacity among patients and the potential role of EHR adoption levels in lowering death rates.

Third, though IT integration and technology adoption are the mainstay of the hospital supply chain system, the systems are by nature endogenous and the possibility of resilience and response to shocks are generally designed into and planned for [70, 71]. COVID-19 pandemic, though a morbid shock, allows us to test for the efficacy of pre-pandemic EHR investments that increasingly are the fabric of interconnectivity among providers in the local ecosystem. By focusing on the level of EHR, we modeled and tested for the potential integration value that EHR integration may provide. We hope that our findings provide some insights into the value of EHR in handling sticky bed capacity limitations during a pandemic.

Fourth, the focus on EHR is important given the informational intensity spikes during a pandemic. Though healthcare supply chain networks are by their very nature information-intensive, the need for improved knowledge exchange and flow is at its highest during a pandemic. Though the pre-pandemic level EHR infrastructure did not account for pandemic needs, we expect that EHR provided the necessary complementarity to manage, if not improve, the information exchange and resource flow overloads.

### Practical implications

The findings of this study provide practical implications for policymakers, hospital management, and stakeholders in the local healthcare ecosystem. With sticky hospital bed capacity, balancing care for COVID-19 and non-COVID-19 patients is a critical goal for hospitals. In doing so, EHR investments could not only help manage the care of non-COVID-19 patients but may also help improve the local flow of patients among hospitals, clinics, and local physicians to manage capacity and provide care. Though a significant body of research has focused on the value of EHR in improving hospital performance, the COVID-19 presents a viable exogenous shock to test whether investments in EHR led to improved management of the relative capacity of COVID-19 and non-COVID-19 hospital beds. Our research question draws on the value of the flow of patients driven by EHR under the exogenous shock of COVID-19.

EHR networks form the essential backbone for developing, implementing, sharing, and leveraging medical data through data-sharing networks and platforms. Though governments across the world have focused on this important investment, it is not uniformly implemented and/or adopted, and its efficacy is increasingly questioned by a variety of stakeholders. Our findings provide support to the value of EHR. Despite the regulatory and logistic burdens of implementing such systems at scale, our findings show that the interoperability of these systems is at the center stage of improving the effectiveness of bed capacity management. With the sudden onset of COVID-19, our findings show that public health investments in EHR, though costly in the short-run can be justified in the long-term.

### Limitations and directions for future research

Our findings are not without limitations. First, we focused on future (7-, 14-, 21-day) death rate outcomes at the county level as our outcome for several reasons. First, the choice of COVID-19 mortality incidence rates at 7th, 14th, and 21st days are based on the incubation and typical mortality rates since the start of infection. Ideally, if reallocation is targeted at providing more beds for COVID cases, and thereby reducing availability of beds for non-COVID cases, a more relevant measure should be the changes in COVID to non-COVID deaths or total deaths. We thank the editor for this suggestion. To our knowledge, the reports of COVID-19 related deaths are more accurately reported, and the mortality rates related to non-COVID-19 rates are not uniformly reported or available and available on a delayed basis (making COVID-19 to non-COVID-19 mortality rates less comparable on similar time scales). Furthermore, different morbidities lead to mortality at a varying pace and therefore, one may overcount or undercount the mortalities from non-COVID-19 deaths during a given period. Given the limitations of our main measure of COVID-19 death rates, an accurate measure of non-COVID-19 death rates at the 7th, 14th, and 21st days would have been more ideal.

Second, because the COVID-19 shock-related response was *in-situ* in the ongoing capacity and the ongoing coordination among local hospitals, management of relative capacity at the hospital level would also spill over into other local hospitals, allowing for improved management, admissions, and care among local hospitals in the local area. Focusing on hospital-level outcomes censors the effects of incoming and outgoing spillovers from managing the relative capacity of beds and the value of EHR percentage that could have ongoing benefits in managing the local ecosystem.

Third, due to local co-dependence, there is strong evidence that hospitals co-opt in the local area to manage the capacity and flow of patients [72], and with increasing, ownership concentration greater coordination among local hospitals is increasingly evident [73]. As such, we expect that greater regional coordination among hospitals, evident in the recent COVID-19 crisis [74, 75], calls for an assessment of regional level improvements in regional incidence rates. Nevertheless, richer theorization and empirical testing based on additional hospital-level micro-data could add further insights.

Fourth, although we control for state effects and conduct a county border pair analysis where many variations in non-pharmaceutical interventions occurred, the intra-regional variations within the counties are not observed. Variations in state and local responsiveness driven by policy differences could be important influencers on the identified association. However, we controlled for state-time effects, analyzed only border counties, and a variety of factors to somewhat assuage this concern for complex and emerging dynamics during a pandemic. Fourth, there is no federal mandate on the COVID-19 bed allocation reporting requirements. However, this voluntary reporting has very high reporting rates, and we expect that the reported data is accurate as there is no incentive to misreport. However, due to the pandemic setting, there may be errors of commission and omission. Though we control for weekly hospital fixed-effects, the variations in stress levels among the staff, shortage of equipment are additional factors to consider for future research. Furthermore, capacity may spillover from less efficient hospitals to more efficient ones. However, the emerging efficiencies in responsiveness cannot be observed. Fifth, there may be non-hospital-related county-level factors that could influence COVID-19 death rates. Though our hospital capacity and non-COVID-19 controls could closely relate to the location, there may be some confounders influencing the outcomes. Finally, our analysis is limited to the US, and the identified relationships may vary in other countries during the pandemic.

### Conclusion

There have been growing calls to understand how hospital supply chains have responded to COVID-19. The response is not easy given the limited ability to expand capacity in the short-run. In this study, we investigated the role relative bed capacity allocation played in lowering local death rates. The simulation and empirical results demonstrate that EHR may be the driver of coordination and integration in an ecosystem under significant pressure in the face of a pandemic. We hope that the findings of this study provide a framework for future research to further study the value of EHR and complementary systems that help lower the incidence of COVID-19 and improve patient care.

## Supporting information

S1 File (DOCX)
