# Supplementary material for: Relative bed allocation for COVID-19 patients, EHR investments, and COVID-19 mortality outcomes
Source: PLoS One. 2023 Oct 26;18(10):e0286210. doi: 10.1371/journal.pone.0286210 (PMC10602360; doi:10.1371/journal.pone.0286210)
Supplement: S1 File — (DOCX) [file pone.0286210.s001.docx]

**SUPPORTING INFORMATION**

**SIMULATION MODEL**

Suppose we have $n$ patients indexed by $i\in\left\{ 1,\ldots,n \right\}.$ The decision problem at the hospital level looks as follows:

|  |  | patient survives |  | patient dies |
| --- | --- | --- | --- | --- |
| COVID-19 patient is allocated a bed |  | $\varpi_{i}$ |  | $1-\varpi_{i}$ |
| COVID-19 patient is not allocated a bed |  | $1-\varphi_{i}$ |  | $\varphi_{i}$ |

The probabilities $\varpi_{i}$ and $\varphi_{i}$ can be approximated using the available EHR percentage in a hospital. The problem is whether patient $i$ should be allocated to a bed ($x_{i}=1)$ or not ($x_{i}=0$). If our purpose is to save as many lives as possible, then we can set the following integer problem:

|  | $\begin{matrix} \max\sum_{i=1}^{n} (\varpi_{i}-\varphi_{i})x_{i}, \\ \sum_{i=1}^{n} x_{i}\leq C, \\ x_{i}\in\{0,1\}, \end{matrix}$ | (1) |
| --- | --- | --- |

where $C$ denotes COVID-19 bed capacity, leading to the analytical solution

|  | $x_{i}=1\Leftrightarrow\varpi_{i}>\varphi_{i}, i=1,\ldots,n.$ | (2) |
| --- | --- | --- |

We perform a simulation experiment with different values of $n,C$ $\varpi_{i}$s and $\varphi_{i}$s. The probabilities $\varpi_{i}$ and $\varphi_{i}$ are drawn randomly from independent standard uniform distributions and also from the distribution function of the standard normal distribution. All experiments are performed 10,000 times.

The inference from Figure A is that based on $\varpi_{i}$s and $\varphi_{i}$s from EHRs, capacity is not reached unless $n>C$. The underlying reason is that hospitals try to use relative bed capacity for COVID-19 patients as efficiently as possible and, possibly, they apply decision theory based on EHR-related probabilities.

FIGURE A: Case types by patients and hospitals

1. **Uniformly distributed probabilities**

**B. Normal probabilities**

Here the probabilities $\varpi_{i}$ and $\varphi_{i}$ are drawn using the distribution function of the standard normal distribution.

FIGURE B: Coordinated [EHR] and uncoordinated [non-EHR] solutions

**The SIR model and Coordinated Solution from EHR across Hospitals**

The basic SIR model in epidemiology is

|  | $\frac{dS}{dt}=-\beta I(t)S(t),$ | (1) |
| --- | --- | --- |
|  | $\frac{dI}{dt}=I\left( t \right)\left[ \beta S\left( t \right)-\gamma-\delta A(t) \right],$ | (2) |
|  | $\frac{dR}{dt}=\gamma I(t),$ | (3) |

where $S,I,R$ denote the number of susceptible people, the number of infected, and the number of recovered persons. Here, $\beta$ is the daily transmission rate, and $\gamma$ is the daily transition rate from infected to recovered (which, so far, seems to be rather close to zero). Moreover, $A(t)$ denotes the median of the distribution of $I(t)$ at period $t$, derived from the integer linear programming problem. In the first difference form, we have:

|  | $S_{t+1}-S_{t}=-\beta I_{t}S_{t},$ | (4) |
| --- | --- | --- |
|  | $I_{t+1}-I_{t}=I_{t}(\beta S_{t}-\gamma-\delta A_{t}),$ | (5) |
|  | $R_{t+1}-R_{t}=\gamma I_{t}+\varepsilon A_{t}.$ | (6) |

It is well known that managing a SIR epidemic means modifying the constants $\beta$ and $\gamma$. We set $\beta=\frac{1}{7} , \gamma=\frac{1}{3}, \delta=\frac{1}{10}, R\left( 0 \right)=0, \varepsilon=\frac{1}{10}, I\left( 0 \right)=0, S\left( 0 \right)=10,000.$

Next, we consider a case with and without coordination. We have 5 regions with 100 patients and a capacity of 200. In Figure B, we show the non-coordinate solutions and the coordinated solution. Although the means are all close to 25% relative to capacity, the coordinated solution has a much lower standard deviation.

Indeed, in the coordinated solution, we see that the curve of infected people over time flattens. The non-coordinated solution corresponds to the solution of the integer linear programming problem with 10 regions each one of which is populated by 1,000 people (Figure C). The simulation demonstrates that a coordinated solution facilitated by EHR may lead to a potentially flatter SIR curve.

FIGURE C: SIR model
